# Patient Perceptions of Sensitive Genetic and Other Medical Information: Findings from a Cancer Survivors Survey in Japan

**DOI:** 10.31662/jmaj.2025-0318

**Published:** 2025-11-28

**Authors:** Mizuho Yamazaki Suzuki, Yuko Ohnuki, Tomoari Mori, Ai Unzaki, Kei Takeshita

**Affiliations:** 1 Department of Medical Ethics, Tokai University School of Medicine, Isehara, Japan

**Keywords:** sensitive medical information, access restrictions, privacy concern, patient-provider discrepancy, stigmatization, medical ethics, electronic medical records (EMRs)

## Abstract

**Introduction:**

Our previous findings indicated that hospitals frequently restrict access to genetic information, while access to other types of sensitive information―such as psychological counseling records or infectious disease diagnoses―is limited in a smaller proportion of facilities (Suzuki et al., 2023). This practice, grounded in a paternalistic medical framework, highlights the need to incorporate patients’ perspectives into future information governance. Therefore, this study aimed to explore how individuals with a history of cancer perceive the sensitivity of various types of medical information, as well as their expectations regarding access control and information sharing.

**Methods:**

We conducted a questionnaire survey among 1,079 cancer survivors, using vignette-style hypothetical scenarios to assess their perceptions of medical information sensitivity and preferences for access restrictions in clinical practice. Participants evaluated 13 types of information that had previously been subjected to restricted access in actual hospitals.

**Results:**

Of the participants, 639 (59.2%) believed that some types of medical information are more sensitive than others. Human immunodeficiency virus (HIV)-related information (64.3%) and information on refractory genetic disorders (57.0%) were most frequently identified as requiring strict access control. Genetic information on hereditary tumors was perceived as significantly less sensitive than that on refractory hereditary diseases (p < 0.05). Only a small fraction of participants believed that such restrictions were unnecessary.

**Conclusions:**

These findings suggest that patients’ perceptions of sensitivity are closely linked to concerns about psychological, social, and ethical vulnerabilities, and may not directly reflect existing institutional access control practices. Notably, patients perceived HIV-related information as particularly sensitive despite limited institutional restrictions, whereas genetic information, though frequently restricted in hospitals, was not always perceived as equally sensitive. This divergence underscores the importance of incorporating both patient and clinician perspectives to align information governance with actual sensitivity concerns. Future studies involving physicians’ perspectives could further elucidate these perceptual disparities and foster more inclusive policymaking in healthcare data management.

## Introduction

The adoption of electronic medical records (EMRs) has been promoted in Japan since 2001, and the current EMR adoption rate in large hospitals with more than 400 beds has reached 93.7% ^(1)^. EMRs serve primarily to facilitate information sharing among healthcare providers, contributing to better care coordination and improved patient outcomes. In addition to these clinical benefits, EMRs are increasingly utilized as a source of big data for research and policymaking, as reflected in national initiatives such as the 2022 Comprehensive Policy, the Medical DX Vision 2030, and legislative measures including the 2018 Next-Generation Healthcare Infrastructure Act ^(2), (3)^.

While EMRs enhance the accessibility and continuity of medical information, there remain situations in which access to certain highly sensitive information is restricted even within healthcare institutions. Our previous studies have shown that information such as genetic data, psychological records, and specific infectious disease information (e.g., human immunodeficiency virus [HIV], coronavirus disease 2019 [COVID-19]) is often subject to restricted access, sometimes being stored separately on paper or electronically protected ^(4)^. However, the criteria for designating such information as “sensitive” are often determined from the perspective of healthcare professionals, and institutional practices vary widely. Our prior work confirmed this variability, showing that approaches to sensitive data management varied markedly across Japanese healthcare institutions ^(4)^.

The Japan Society of Health Information Management Guidelines recommend that healthcare institutions define categories of highly confidential information, including genetic information and clinical trial data, and apply appropriate access restrictions in accordance with institutional policies ^(5)^. By contrast, in other countries, legal frameworks provide explicit protections for sensitive medical information. In the United States, the Health Insurance Portability and Accountability Act establishes specific protections for mental health and HIV-related data, while in the European Union, the General Data Protection Regulation (GDPR) classifies health and genetic data as “special categories of personal data” requiring enhanced safeguards ^(6), (7)^. In this study, we defined “sensitive” medical information as data that, if disclosed inappropriately, could cause personal, social, psychological, or economic harm to patients or their families. This definition aligns with the concept of “special categories of personal data” under the European Union GDPR, which includes health data, genetic data, and data concerning a person’s sex life or sexual orientation ^(7)^.

However, little is known about how patients perceive which types of medical information are sensitive and warrant access restrictions. This gap is particularly important in Japan, where no specific legal framework exists and institutional practices rely on professional guidelines. Moreover, as health data are increasingly used in national big data initiatives under the Next-Generation Healthcare Infrastructure Act, and as Japan gradually advances toward broader electronic health record integration, where patients may eventually gain access to their records, incorporating patient perspectives will be critical to ensuring fairness, trust, and equitable secondary use of data. To better balance effective information sharing with appropriate privacy protections, it is necessary to understand how patients themselves perceive the sensitivity of their medical information.

In this study, we surveyed individuals with a history of cancer to explore which types of medical information they perceive as highly sensitive and requiring access control. Cancer survivors were chosen because, in Japan―where one in two people are affected during their lifetime―they represent not only a population with repeated healthcare experiences, but also citizens whose perspectives offer valuable insights into the social dimensions of data sensitivity. We also assessed their preferences regarding who should determine access rules for such information.

## Materials and Methods

In this study, we used vignette-style hypothetical scenarios to explore which types of information cancer survivors perceive as sensitive and subject to restricted access in Japanese hospitals. This approach was chosen to simulate realistic clinical situations and elicit participants’ perceptions in a concrete and relatable manner.

The specific scenarios used in the survey are shown below (Table 1).

**Table 1.** Vignette-Style Hypothetical Questions were Used to Assess Participants’ Perceptions of Access Restrictions to EMRs.

No	Hypothetical scenario
Q1	You have been diagnosed with appendicitis.
Q2	You have been diagnosed with hereditary cancer.
Q3	You have been diagnosed with HIV.
Q4	You have experienced domestic violence and disclosed it to your doctor.
Q5	Your child has been found to carry a hereditary cancer risk, but has not yet developed the disease.

Participants were presented with the following hypothetical scenarios and were asked the same question for each: “In this situation, should access to your EMR be restricted?” Responses were recorded using a 5-point Likert scale (1 = Not at all necessary to 5 = Very necessary). EMR: electronic medical record.

The vignettes were iteratively reviewed and refined by coauthoring physicians and experts in medical ethics to ensure their validity, clinical relevance, and ethical appropriateness.

The study was conducted as a questionnaire-based survey targeting 1,079 cancer survivors, recruited through an online research panel. The target sample size was set at approximately 1,000 participants to facilitate potential future comparisons with a similarly designed physician survey. In this study, “cancer survivors” were defined in accordance with the international standard, encompassing individuals currently receiving treatment, under follow-up, or considered cured. Cancer survivors were selected because they tend to have repeated interactions with healthcare providers over time, allowing access to a large sample with relatively shared clinical experiences. Moreover, in Japan, cancer has historically been regarded as sensitive information, which may position cancer survivors to reflect on the personal and social implications of medical data sensitivity. Notably, during the 1990s, cancer was often considered too emotionally distressing to disclose directly to patients and was treated as highly sensitive even within families and clinical settings. However, this period preceded the widespread implementation of EMRs, and structured access control systems for sensitive data were not yet established.

The questionnaire was developed by the research team, while the creation of the survey website and data collection were outsourced to an online survey company (Intage Co., Ltd.). Participants were selected from Intage’s panel of general citizens with cancer experience (cancer survivors). The survey was conducted over a 5-day period from February 16 to 20, 2024, and recruitment was closed upon reaching the target sample size. Prior to participation, the study purpose was explained via an introductory statement on the survey website, and only individuals who read the explanation and provided online informed consent were included. Participants received compensation from Intage Co., Ltd. in the form of reward points. All responses were anonymized before being provided to the researchers.

The questionnaire included items on demographic characteristics (such as age, gender, family structure, and type of hospital attended) and health-related variables (including years since diagnosis and current treatment status). Participants were also asked to estimate which categories of healthcare staff they believed had access to all information stored in EMRs.

In addition, participants answered vignette-style hypothetical questions about whether specific types of information should be subject to access restrictions within EMRs. Prior to answering, participants were informed that EMRs are normally designed to facilitate information sharing among authorized medical staff, while some medical institutions impose exceptional access restrictions for particularly sensitive information. For the purposes of this survey, participants were asked to consider more detailed or department-specific restrictions (e.g., limiting access to certain departments or clinicians, such as restricting internal medicine from viewing records when only surgical care was provided). They were also asked about their experiences disclosing sensitive information to healthcare providers and their views on how such information should be managed within EMRs.

Responses were recorded using a 5-point Likert scale. The vignette-style questions (Q8-Q21) used this 5-point scale with one response permitted per item. Among the background items, Q7 allowed multiple responses, whereas Q26 required respondents to select a single option (choosing the most frequent category if multiple applied). The questionnaire contained 27 items and required approximately 13 minutes to complete.

Statistical analyses were performed using Fisher’s exact test in R software (version 4.1.2). Statistical significance was defined as p < 0.05. Pairwise comparisons were conducted between selected vignette items to evaluate differences in participants’ preferences regarding access restrictions. Odds ratios (ORs) and corresponding *p* values were calculated to evaluate the magnitude and statistical significance of these differences.

The study protocol was approved by the Tokai University Institutional Review Board for Human Research (approval number 23153).

## Results

We received responses from 1,079 cancer survivors. There were 591 female and 488 male participants, with a mean age of 59 (±12.1 years). Regarding family structure, 881 participants lived with their families, 193 lived alone, and five reported other living arrangements.

The types of medical institutions attended included general hospitals (55.3%), university hospitals (23.4%), cancer hospitals (12.2%), and clinics or medical practices (6.9%). Regarding treatment status, 50.3% of participants were under observation, 47.7% were receiving active treatment, and 1.9% reported being completely cured. The time since diagnosis ranged from less than one year (12.1%) to 10 years or more (10.7%) (Table 2).

**Table 2.** Participant Demographic and Clinical Characteristics (n = 1,079).

Variable	Category	n	%
Sex	Male	488	45.2%
	Female	591	54.8%
Age, mean SD, y	59(±12.1)	−	
Household composition	Living with family	881	81.6%
	Living alone	193	17.9%
	Other	5	0.5%
Medical facility type*	General hospital	597	55.3%
	University hospital	253	23.4%
	Cancer hospital	132	12.2%
	Clinic or medical practice	74	6.9%
	Other	23	2.1%
Treatment status	In observation	543	50.3%
	Undergoing treatment	515	47.7%
	Completely cured	21	1.9%
Years since diagnosis	<1 year	131	12.1%
	1-3 years	391	36.2%
	3-5 years	207	19.2%
	5-10 years	235	21.8%
	≥10 years	115	10.7%

＊The asterisk indicates the type of medical facility most frequently visited for cancer treatment.

### Patient perceptions of EMR access

Participants were asked which professionals or personnel in the hospital they believed had full access to the EMRs. The responses were as follows: 98.6% identified doctors, 63.6% nurses, 54.1% pharmacists and laboratory technicians, 24.6% administrative staff, 24.8% medical researchers, 15.3% EMR vendors, and 15.1% certified anonymized data processing vendors (Table 3).

**Table 3.** Participant Perceptions of Staff Access to All Information in EMRs (n = 1,079).

Profession/Staff	n	%
Doctors	1064	98.6%
Nurses	686	63.6%
Pharmacists, etc.*	584	54.1%
Administrative staff	266	24.6%
Medical researchers	268	24.8%
EMR vendors	165	15.3%
Certified anonymized data processing vendors	163	15.1%

＊Pharmacists, etc.: This refers to healthcare professionals other than doctors and nurses, such as pharmacists, radiology technicians, and physiotherapists. EMR: electronic medical record.

### Vignette-style hypothetical questions

**Figure 1.**
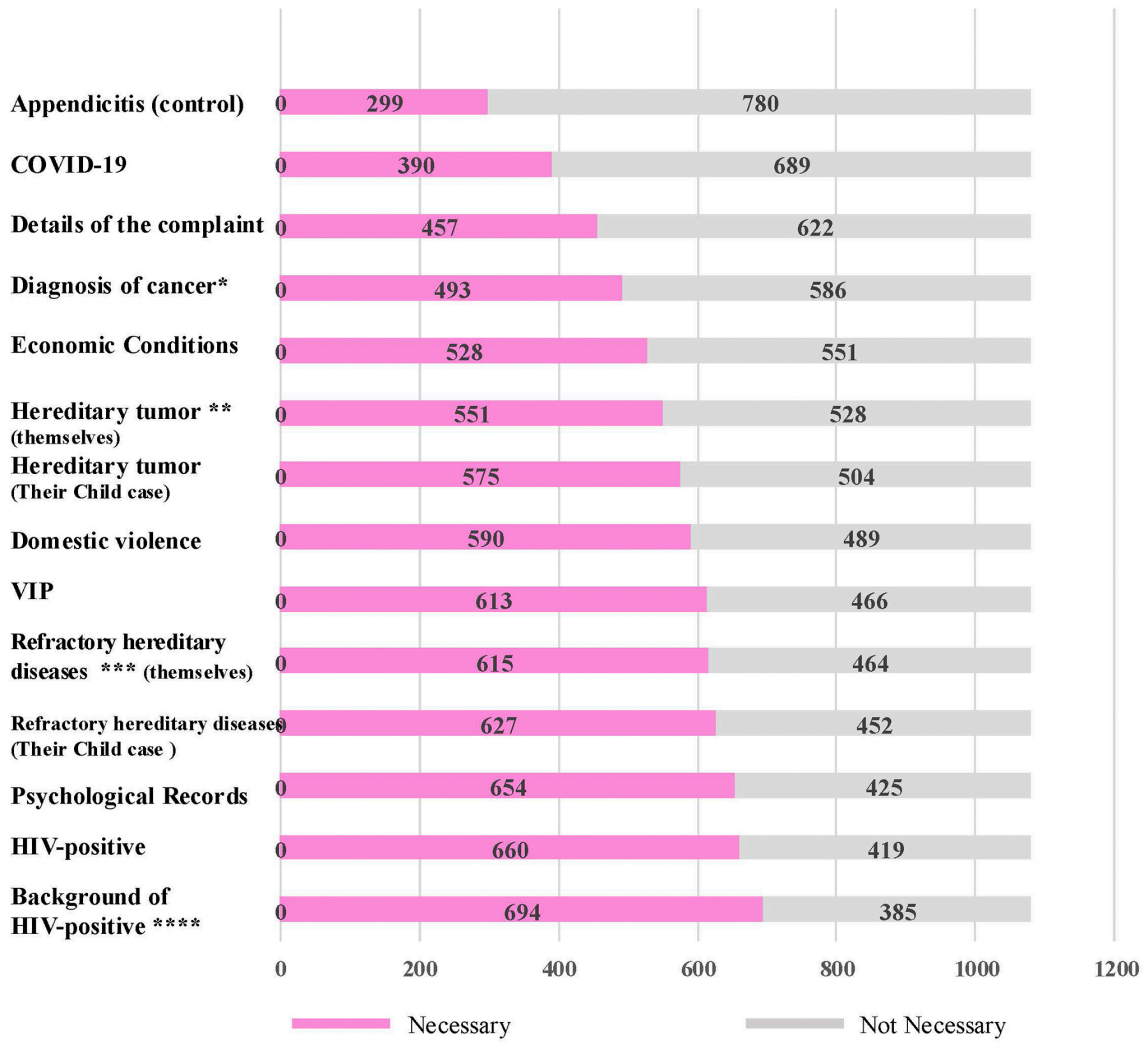
Rates of items requiring access restrictions based on vignette-style hypothetical questions (n = 1,079). Participants were presented with a series of hypothetical clinical scenarios and asked whether access restrictions were necessary for each type of information. Response options included: “very necessary,” “necessary,” “neither necessary nor unnecessary,” “not necessary,” and “not at all necessary.” Responses of “very necessary” and “necessary” were combined into the “necessary” category shown in pink. Statistical comparisons were made against control items as noted below: * _p_ < 0.001 vs. appendicitis (control); OR = 2.19 ** _p_ = 0.014 vs. cancer diagnosis; OR = 1.24 *** _p_ = 0.006 vs. hereditary tumor (self); OR = 1.27 **** _p_ < 0.001 vs. hereditary tumor (self); OR = 1.73 OR: odds ratio.

Participants were asked whether they preferred access restrictions for 14 items presented as vignette-style scenarios. Of these, 12 items had been identified in a previous study as potentially sensitive medical information ^(4)^. In the present study, the diagnosis of cancer was newly added due to its direct relevance to the participant population and its historical classification in Japan as highly sensitive information, particularly prior to the widespread implementation of EMRs. Additionally, information on appendicitis―generally not considered sensitive in clinical practice―was included as a control item. Responses were collected on a 5-point Likert scale: “highly necessary,” “necessary,” “neither,” “unnecessary,” and “completely unnecessary.” Responses of “highly necessary” and “necessary” were combined into a single “necessary” category (Figure 1).

For all 13 items, the proportion of participants who indicated that access restrictions were necessary was significantly higher than for appendicitis.

Specifically, 45.7% of participants supported restricting access to their cancer diagnosis, compared to 27.7% for appendicitis (p < 0.001, OR = 2.19). A significantly higher proportion also supported restricting access to information on hereditary tumors (51.1%) compared to cancer diagnoses (p = 0.014, OR = 1.24). However, there was no statistically significant difference in the desire to restrict access to genetic information on hereditary tumors between participants’ own pathogenic variants (51.1%) and those of their unaffected children (53.3%) (p = 0.322, OR = 1.09). Additionally, participants were significantly more likely to prefer access restrictions for refractory hereditary diseases (57.0%) than for hereditary tumors (51.1%) (p = 0.006, OR = 1.27). HIV-related information received the highest proportion of responses, indicating that access restrictions were necessary (64.3%). This proportion was significantly higher than that for hereditary tumors (p < 0.001, OR = 1.73), underscoring the exceptional sensitivity associated with HIV-related data among participants.

A full comparison of responses across all vignette items is presented in Figure 1.

### Concerns regarding the use of medical information

**Figure 2.**
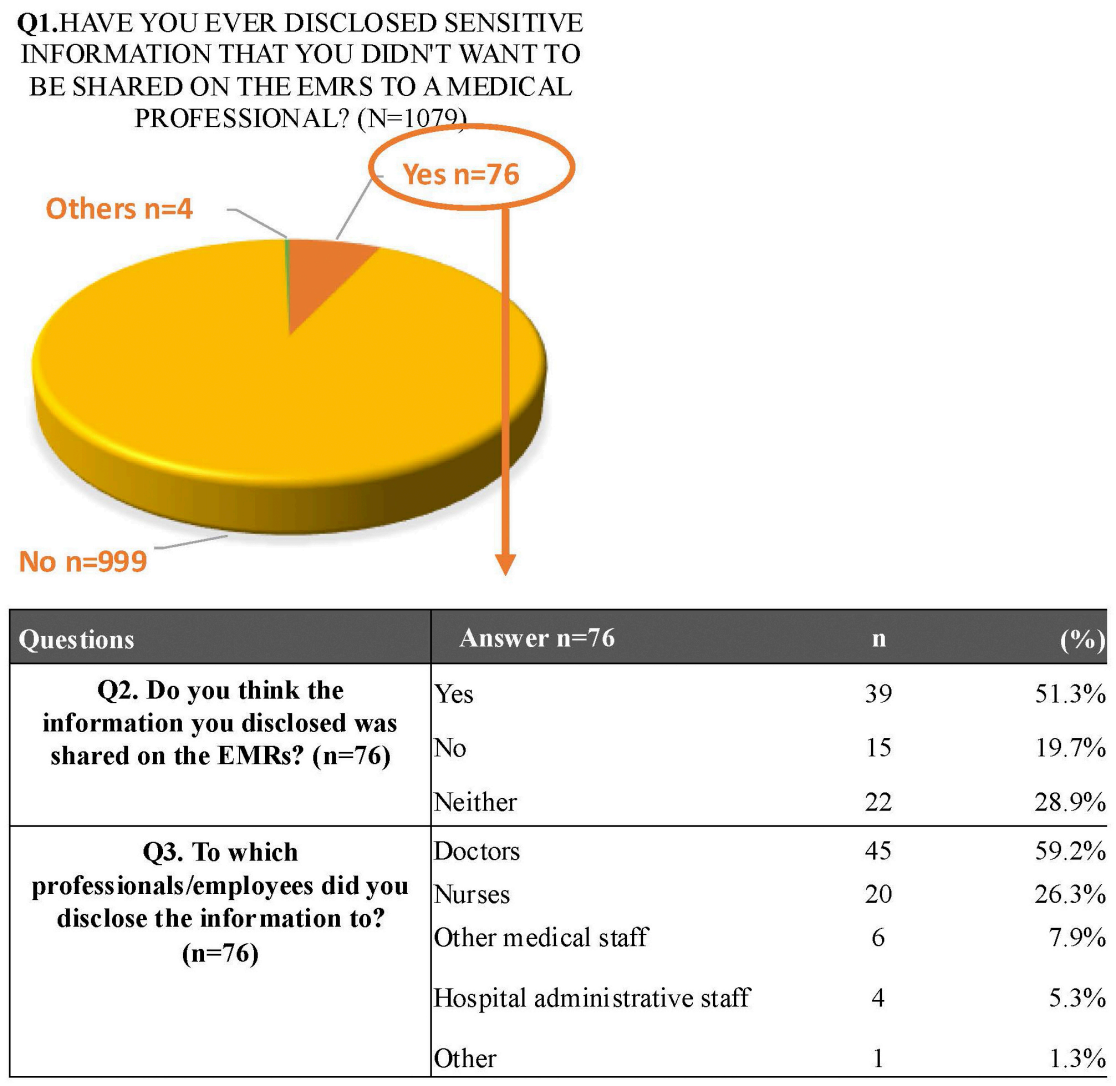
Participants’ experiences of disclosing sensitive information, recipients of the disclosure, and perceived inclusion in the EMRs. Participants reported whether they had ever disclosed information they preferred not to be widely shared in the EMRs, to whom they disclosed it, and whether they believed it had been documented. The content of the information was not specified. EMR: electronic medical record.

Participants were asked whether, as patients, they had ever disclosed sensitive information to healthcare providers within the hospital, and whether they believed that such information had subsequently been recorded in their EMRs. Seventy-six participants (7.0%) reported having such experiences, while the majority indicated they had not (Figure 2).

Among these participants, 59.2% disclosed the information to physicians, 26.3% to nurses, 7.9% to other healthcare professionals, 5.3% to administrative staff, and 1.3% to others.

When asked whether they believed that the sensitive information they had disclosed had been documented in the EMRs, 39 participants (51.3%) responded affirmatively, 22 (28.9%) were uncertain, and 15 (19.7%) believed it had not been documented.

### Perceived sensitivity and patient preferences for information handling

A total of 639 participants (59.2%) responded that medical information contains highly confidential content and that sensitivity levels vary depending on the type of information. In contrast, 321 participants (29.7%) answered “neither,” and 119 (11.0%) responded that there is no variation in sensitivity levels.

**Figure 3.**
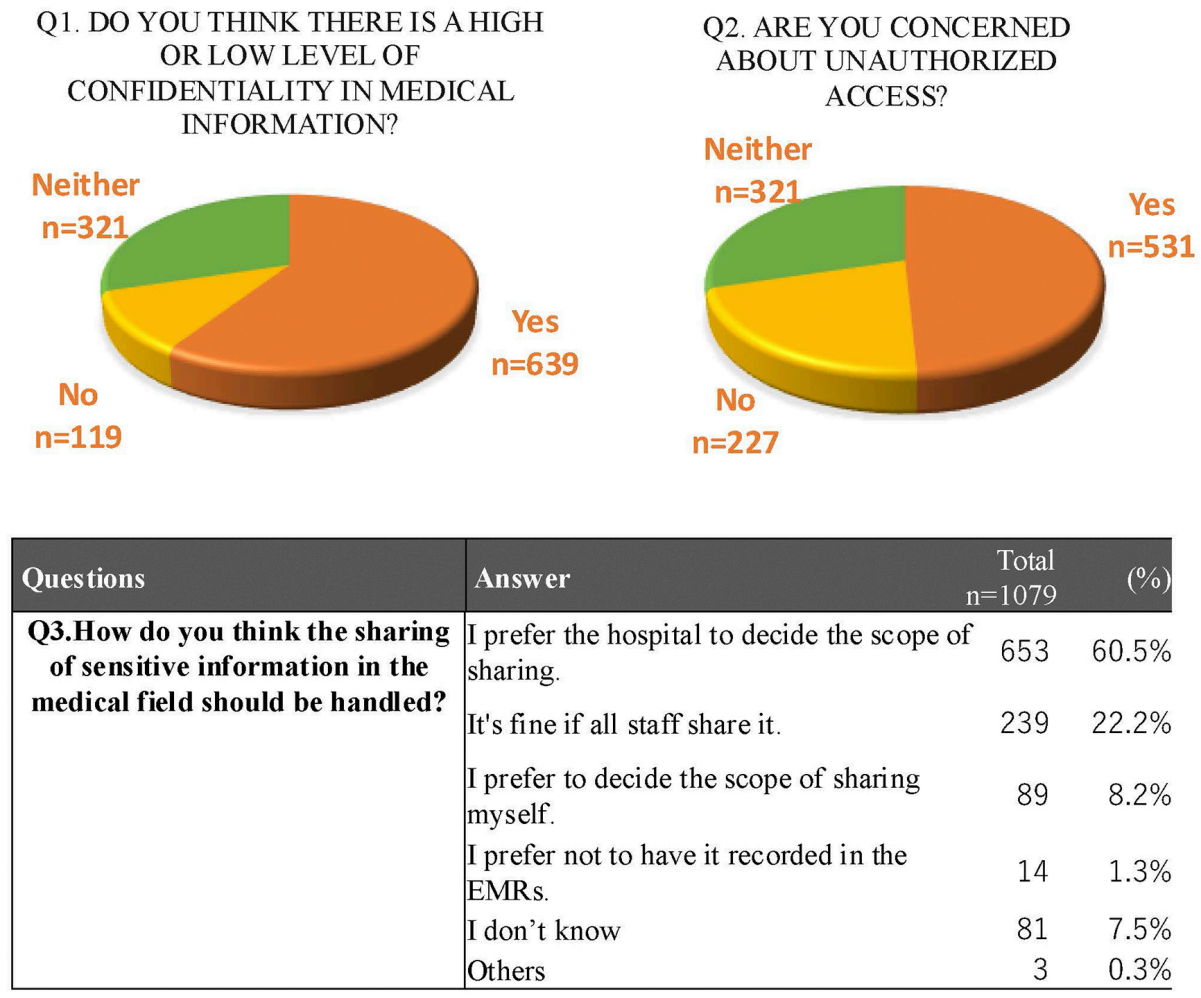
Participants’ preferences and concerns regarding the management of sensitive information. Participants responded to questions about confidentiality levels, concerns over unauthorized access, and preferences for information-sharing practices in clinical settings.

When asked about concerns regarding unauthorized access to EMRs, 531 participants (49.2%) reported being concerned, 320 (29.7%) were neutral, and 226 (21.0%) indicated no particular concern (Figure 3).

Participants were also asked about their preferences for the handling of highly confidential information that may arise in medical care. The majority (60.5%) preferred that the hospital determine the scope of information sharing. In comparison, 8.2% preferred to decide the scope themselves. Additionally, 22.2% were comfortable with sharing such information with all staff members, while 1.3% indicated that they did not want such information recorded in EMRs.

## Discussion

### Overview of perceived sensitivity

In this study, participants with a history of cancer demonstrated varied perceptions of the sensitivity of different types of medical information. Overall, participants considered certain types of information to warrant access restrictions more than others. HIV-related information was most frequently perceived as highly sensitive, followed by psychological records, genetic information on refractory hereditary diseases, and genetic information on hereditary tumors. In contrast, general medical information, such as appendicitis, was rarely seen as requiring special access controls (Figure 1).

These findings suggest that patients distinguish between different types of medical information based not only on clinical content but also on the perceived potential for personal, social, or psychological harm if the information were widely accessible. Such potential harms are not merely hypothetical. For example, the Ministry of Health, Labour and Welfare has documented the so-called “AIDS panic” of the mid-1980s in Japan, when people living with HIV faced stigma and discrimination, including exclusion from public facilities. In direct response, HIV antibody testing at public health centers was offered free of charge and on an anonymous basis ^(8), (9)^, a uniquely Japanese system that reflects societal recognition of the risks associated with disclosure. In the following sections, we examine (1) the divergence between genetic information and HIV, and (2) the distinct stigma mechanisms in HIV and COVID-19.

### Divergence between genetic information and HIV

Interestingly, while medical institutions have often prioritized genetic information for access restrictions in clinical practice, patients in this study perceived HIV-related information as even more sensitive. Previous surveys of healthcare institutions have shown that many hospitals restrict access to genetic information due to concerns about the psychological, social, and ethical consequences for patients ^(4), (10)^. Genetic information is often associated with stigma, not only for the affected individuals but also for their family members, due to its implications for hereditary risk.

This contrast between institutional focus and patient perceptions may reflect differences in knowledge and risk awareness. Healthcare professionals, possessing specialized knowledge in medical genetics and ethics, are acutely aware of the potential for genetic information to lead to discrimination in contexts such as marriage, employment, and insurance. Consequently, many institutions have implemented stringent access restrictions on genetic data within EMRs to prevent unintended disclosure and protect patients from possible harm ^(10)^.

In contrast, patients may be more strongly influenced by direct or indirect personal experiences or media narratives surrounding stigma and discrimination, particularly those related to HIV. In Japan, HIV-related discrimination has historically been linked to assumptions about personal lifestyle, sexual minorities, and marginalized populations, leaving a vivid and lasting impression among patients ^(11), (12)^. This interpretation is consistent with established stigma literature, which has long documented how illnesses such as HIV and other infectious diseases are linked to presumed personal characteristics or moral failings, leading to social discrediting ^(13), (14)^. This divergence highlights the importance of re-evaluating access policies to incorporate both professional assessments of medical risk and patients’ lived experiences and concerns.

### Distinct stigma mechanisms in HIV and COVID-19

The divergent perceptions of sensitivity regarding HIV and COVID-19 observed in this study further illuminate the distinct mechanisms underlying medical stigma. Although both conditions have led to social exclusion and discrimination, their sources and persistence differ. In the case of HIV, stigma has long been associated with behaviors that are often morally charged, such as sexual orientation, sexual practices, and drug use, leading to deeply rooted structural and cultural stigma ^(15)^. Individuals may be judged not only for their infection status but also for presumed personal characteristics or moral failings.

By contrast, COVID-19 stigma primarily arose from fear of contagion and public anxiety during the early phases of the pandemic. Although discrimination against infected individuals, healthcare workers, and their families was widely reported, COVID-19 was generally framed as a public health crisis rather than as a moral issue ^(16), (17), (18), (19)^. Over the four years since the onset of the pandemic, public perceptions of COVID-19-related sensitivity have markedly declined.

The contrast between HIV and COVID-19 highlights how moralized narratives continue to sustain HIV-related stigma even today, whereas the sensitivity associated with COVID-19 has diminished as its association with personal responsibility has weakened. These findings suggest that the enduring sensitivity surrounding HIV in Japanese society may reflect persistent cultural narratives linking infection to personal identity and morality.

### Broader implications for access control and future directions

Building on these findings, patients also expressed a need to restrict access to personal information beyond strictly medical or biological domains. These included information about financial circumstances, psychological state, experiences of domestic violence, and other sensitive aspects of their private lives. Such concerns reflect patients’ broader expectations regarding privacy and dignity in the clinical setting. Despite this, only 7% of participants reported voluntarily disclosing highly sensitive personal information to medical staff, and approximately half believed that such information had been documented in EMRs. Most participants preferred that healthcare institutions take responsibility for determining and managing access to highly sensitive information.

While this study examined patients’ perspectives using hypothetical scenarios, future research should incorporate the viewpoints of healthcare professionals―particularly physicians involved in cancer care―to examine potential discrepancies in sensitivity perceptions and promote better alignment in information governance and institutional policies.

### Study limitations

This study has several limitations. First, it was conducted among approximately 1,000 individuals who had been diagnosed with cancer and may not fully reflect the perceptions of the general patient population in Japan. Although we attempted to balance the sample by sex and age group, the number of young participants was inevitably small; therefore, age was presented as a mean rather than by detailed strata. In addition, we did not stratify by cancer type, and cancer-type-specific stigma may have influenced participants’ responses; this potential bias should be considered when interpreting the findings. Furthermore, because the survey was conducted online, selection bias is possible, as relatively healthier patients may have been more likely to participate. Prior experiences with infectious disease testing, as well as the universal impact of the COVID-19 pandemic, may also have shaped participants’ perceptions of sensitivity. Second, the survey relied on hypothetical scenarios to assess participants’ attitudes, which may not accurately reflect actual behaviors or preferences in real-world clinical situations. Third, as the study was based on self-reported data, there is a possibility of recall bias or social desirability bias influencing participants’ responses. Fourth, the study focused exclusively on patients’ perspectives; future research should include healthcare professionals, particularly physicians involved in cancer care, to compare and clarify potential differences in perceptions. Lastly, since this study was conducted within Japan’s healthcare system, the findings may not be directly generalizable to other countries with different healthcare infrastructures and cultural norms.

### Conclusions

Unlike previous studies that primarily emphasized genetic information as highly sensitive, our study revealed that patients perceived HIV-related information as even more sensitive than genetic data. This divergence underscores the enduring influence of stigma and discrimination surrounding HIV in Japan. These findings highlight that information governance should not be based solely on biomedical considerations but must also incorporate patients’ lived experiences and social context. A key future challenge will be to integrate these differing perspectives of patients and healthcare professionals into governance frameworks and medical policy.

## Article Information

### Acknowledgments

We sincerely thank all the cancer survivors for their valuable time and effort in responding to the questionnaires.

### Author Contributions

Contributed to the study conception and design: all authors. Material preparation, data collection, and analysis: Mizuho Yamazaki Suzuki, Yuko Ohnuki, and Kei Takeshita. First draft of the manuscript: Mizuho Yamazaki Suzuki and all authors commented on previous versions of the manuscript. Read and approved the final manuscript: all authors.

### Conflicts of Interest

None

### IRB Approval Code and Name of the Institution

This study was approved by the Tokai University Institutional Review Board for Human Research (23153).

## Supplement

Supplementary Material
