# Supplementary material for: Patient Perceptions of Sensitive Genetic and Other Medical Information: Findings from a Cancer Survivors Survey in Japan
Source: JMA J. 2025 Nov 28;9(1):141–9. doi: 10.31662/jmaj.2025-0318 (PMC12889046; doi:10.31662/jmaj.2025-0318)
Supplement: Supplementary Material [file 2433-3298-9-1-0141-s001.pdf]

Supplementary Material Questionnaire (English Version)

Study Title: Questionnaire on the Management of Electronic Medical Records

Principal Investigator: Mizuho Yamazaki Suzuki, Department of Medical Ethics, Tokai University School of Medicine

Introduction:

In medical institutions, medical records are created during patient visits. While paper records were predominantly used in the past, electronic medical records (EMRs) have become increasingly common. EMRs are typically accessed with individual staff passwords, allowing medical personnel to view patient information from any terminal within the facility. Although EMRs enhance the convenience of sharing information for treatment and care, our research has found that nearly half of medical facilities restrict access to particularly sensitive information, even among staff. This is referred to as "access restrictions."
Balancing the sharing and confidentiality of medical information is crucial. We ask for your views on how you would like medical information to be managed.
Estimated time: Approximately 13 minutes
* The results will be aggregated and may be published as statistical data in academic conferences or journals. Once responses are submitted, they cannot be withdrawn.

If you agree to the above, please click "Next" to proceed with the questionnaire.

Questions:

Q1 Please indicate your gender.

Q2 Please indicate your age.

Q3 Please describe your family structure. Do you live with any family members?

Q4 Please describe your current health status.

Q5 How many years have passed since your cancer diagnosis?

Q6 Please select the primary medical institution where you are (or were) receiving cancer treatment.
1) University hospital 2) General hospital 3) Cancer center 4) Local clinic 5) Other

Q7 Which categories of hospital staff do you think have full access to your EMR? (Select all that apply)
1) Physicians 2) Nurses 3) Pharmacists or laboratory technicians 4) Medical researchers 5) Administrative staff 6) EMR system vendors 7) Certified anonymized data processors (authorized by the Ministry of Health for big data collection)

Vignette-Based Scenarios (5-point scale: Strongly necessary to Not necessary):

Before answering the following hypothetical scenarios (vignettes), please read the following explanation:

Medical information is shared through electronic medical records (EMRs) to support treatment and care. Unauthorized viewing of medical information by staff without clinical involvement is considered improper access and is strictly prohibited. Therefore, please assume that such improper access does not occur in these hypothetical situations. In each scenario, please consider whether you believe certain "highly sensitive information" ("kibi-na information") should not be widely shared among medical staff.

Definitions:

- Highly sensitive information: Specific medical information that you believe should not be widely shared with many medical staff via EMRs.
- Access restriction: Limiting EMR access so that staff not directly involved in treating the particular condition cannot view that information (e.g., records from internal medicine are not accessible to surgical departments unless necessary).

Q8 You were diagnosed with appendicitis and hospitalized in the gastrointestinal surgery ward. Do you think special access restrictions are necessary for this diagnosis?

Q9 You visited a psychiatrist and shared your concerns, which were recorded. Do you think special access restrictions are necessary for these psychological records?

Q10 You were diagnosed with COVID-19 and hospitalized. Do you think special access restrictions are necessary for these test results?

Q11 You were diagnosed with HIV infection after examination. Do you think special access restrictions are necessary for these test results?

Q12 Your physician inquired about the reason for your HIV infection. Do you think special access restrictions are necessary for the reasons related to your HIV infection?

Q13 Genetic testing revealed you carry a mutation associated with an untreatable hereditary disease. Do you think special access restrictions are necessary for these genetic test results?

Q14 The same hereditary mutation was found in your child, who has not yet developed any symptoms. Do you think special access restrictions are necessary for your child’s genetic test results?

Q15 You were diagnosed with cancer following a gastrointestinal examination. Do you think special access restrictions are necessary for this diagnosis?

Q16 Genetic testing revealed your cancer is hereditary. Do you think special access restrictions are necessary for these genetic test results?

Q17 Your child was found to carry the hereditary cancer gene, indicating a high risk of developing cancer in the future, although currently healthy. Do you think special access restrictions are necessary for your child's genetic test results?

Q18 You are dissatisfied with hospital policies and wish to file a complaint. Do you think special access restrictions are necessary for the contents of your complaint?

Q19 You discussed financial difficulties related to medical expenses with the hospital. Do you think special access restrictions are necessary for this financial information?

Q20 You visited the hospital due to domestic violence. Do you think special access restrictions are necessary for this information?

Q21 You are a famous celebrity. Do you think special access restrictions are necessary for your EMR information?

Following the vignettes, please provide your opinions on the following questions:

Q22 Do you think there are varying degrees of sensitivity even within protected medical information? (5-point scale)

Q23 Are you concerned about unauthorized access of your EMR? (5-point scale)

Q24 Have you ever disclosed highly sensitive information to medical staff that you preferred not to share widely within the hospital?
1) Yes 2) No 3) Other

Q25 If you answered yes to Q24, do you think this highly sensitive information was recorded in your EMR? (5-point scale)

Q26 To which type of medical staff did you disclose the highly sensitive information? (Choose one; if multiple, select the most frequent)
1) Physician 2) Nurse 3) Other healthcare worker 4) Administrative staff 5) Other

Q27 How would you like highly sensitive information to be shared within the hospital?
1) The hospital should regulate the range of staff sharing the information.
2) Since it is part of the medical record, it may be shared with all staff.
3) I want to decide who can access it.
4) I do not want it recorded in the EMR.
5) Other
6) I do not know

This supplementary file is provided to describe the full set of questionnaire items used in the study.

＜題＞電子カルテ上の情報管理についてのアンケート

病院などの医療施設を受診すると、診療の記録は「カルテ」に記載されます。かつては紙に直接記載する紙カルテの使用が大半でしたが、近年はコンピューター上に記録する「電子カルテ」の利用が増えています。
電子カルテには職員の個別のパスワードで入る仕組みが一般的です。医療スタッフが院内のどこにいても、電子カルテの端末から患者さんの様子を見て、医療に活かせる利便性があります。
ところが、患者さんの特別な（機微な）データを閲覧する時にだけ、職員であってもアクセスできないように制限を設けている医療施設が半数近くあるということが私たちの調査でわかりました。ここではカルテ閲覧の特別な制限をアクセス制限と呼んでいます。
医療データは医療スタッフで共有して治療やケアに活かせることが重要ですが、どこまで電子カルテで共有して良いのか悩む情報もあります。そこで医療情報をどのように管理してもらいたいと思っているのかを教えてください。
所要時間約 13 分

研究実施責任者　東海大学医学部基盤診療学系 医療倫理学　鈴木みづほ

＊結果は数値化し、統計データとして学会や論文等で公表される可能性がありますが、回答後に回答の撤回はできません
上記内容に同意される方は「次へ」ボタンを押して、アンケートにお進みください

Q1 あなたの性別をお答えください。
Q2 あなたの年齢をお答えください。
Q3 あなたの家族構成を教えてください。同居の家族はいらっしゃいますか。
Q4 あなたの現在の健康状態について教えてください。
Q5 あなたのがんが診断されてから、何年経過しているかを教えてください。
Q6 がんの治療に通っている（もしくは通っていた）主な医療施設を 1 つお答えください。(単一回答)
１）大学病院２）一般の総合病院３）がん専門病院４）近医のクリニック５）その他
Q7 あなたの電子カルテを全て見ることができる権限（閲覧権限）は、病院のどのような職種のスタッフが持っていると思いますか。持っていると思う職種全てにチェックをつけてください。(複数選択可)
１）医師　２）看護師　３）薬剤師や検査技師　４）医学研究者　5)病院の事務員　6）

電子カルテシステムの業者　7）匿名加工業者*1
＊1 電子カルテからビッグデータを収集するため、厚労省から認定を受けた業者

ここから架空の場面を記載した文章（ビネット）を読んでいただき、その場面でのあなたの考えを教えてください

医療情報は電子カルテで共有して、治療やケアに活かす重要な情報です。医療スタッフが診療と関係のない情報を興味本位に閲覧することは不正閲覧と言われ、禁じられていますので、この架空の場面でもそのような不正な閲覧や利用はないと思って答えてください。あなたは多くの医療スタッフで共有すべきではない「機微な情報」はあると考えますか？

＜用語の定義＞

機微な情報とは

多くの医療スタッフによって電子カルテで共有しない方が良いと思われる特別な医療情報

アクセス制限とは

このセクションの質問で想定している「特別な電子カルテのアクセス制限」とは、直接その疾患で診療を行っている医療従事者以外には電子カルテを閲覧できないように設定することです。たとえば、特段の事情がない限り、内科で受けている診療の記録を外科では閲覧できないようにすることを想定しています。

アクセス制限が大いに必要、から全く必要なしの5段階評価を実施（Q 8 ～21 まで）

Q8 あなたは虫垂炎（盲腸）となり、消化器外科病棟に入院しました。この診断結果について、特別な電子カルテのアクセス制限は必要だと思いますか。

Q9 あなたは心のバランスを崩し、精神科に受診して悩んでいることを話しました。あなたの悩みを記した心理記録について、特別な電子カルテのアクセス制限は必要だと思いますか。

Q10 あなたは発熱で、内科を受診したところ、新型コロナウイルス感染症と診断され入院しました。この検査結果について、特別な電子カルテのアクセス制限は必要だと思いますか。

Q11 あなたはだるさが続いたので、内科を受診し精査したところ、HIV ウイルス感染症と診断されました。この検査結果について、特別な電子カルテのアクセス制限は必要だと思いますか。

Q12 あなたの HIV ウイルス感染に至った理由を内科の医師は尋ねました。HIV ウイルス感染の理由について、特別な電子カルテのアクセス制限は必要だと思いますか。

Q13 遺伝子検査の結果は陽性であり、あなたは生まれつき病気になる遺伝子の変化を有することがわかりました。この病気は治療法がない遺伝性の病気です。この遺伝子検査の結果に特別な電子カルテのアクセス制限は必要だと思いますか。
Q14 治療法がない遺伝性の病気の遺伝子の変化はあなたの子も有する（陽性）ことがわかりました。あなたの子はまだ病気の症状はありません。子の遺伝子検査の結果に特別な電子カルテのアクセス制限は必要だと思いますか。
Q15 あなたは胃痛で消化器内科を受診し、がんであることがわかりました。この診断結果に特別な電子カルテのアクセス制限は必要だと思いますか。
Q16 あなたの遺伝子検査の結果、がんは遺伝性のがんであると診断を受けました。この遺伝子検査の結果に特別な電子カルテのアクセス制限は必要だと思いますか。
Q17 あなたの子も、あなたと同じように病院で検査した結果、遺伝性のがんの遺伝子が明らかになり、将来がんを発症する可能性が高いとわかりました。子は現在健康です。子の遺伝子検査の結果に特別な電子カルテのアクセス制限は必要だと思いますか。
Q18 あなたは病院の方針に不満があり、クレームを入れたいと思っています。あなたのクレームの内容は特別な電子カルテのアクセス制限は必要だと思いますか。
Q19 あなたは医療費の支払いに困っていることを病院で相談しました。あなたの経済状況の内容について特別な電子カルテのアクセス制限は必要だと思いますか。
Q20 あなたは家族からの暴力で病院を受診しました。家族からの暴力について特別な電子カルテのアクセス制限は必要だと思いますか。
Q21 あなたは有名なスターです。あなたのカルテの情報について特別な電子カルテのアクセス制限は必要だと思いますか。
以上で架空質問は終了です　以下よりあなたの考えを教えてください

Q22 もともと医療情報はすべて要配慮個人情報であり、電子カルテシステムは許されたスタッフだけが入れる仕組みとなっています。その医療情報の中にも秘匿性の高低、つまり機微の程度の差はあると思いますか。 ここからは、あなたの考えを教えてください（５段階評価を実施）。

Q23 医療スタッフが診療と関係のない医療情報を興味本位に閲覧することは不正閲覧と言われ、禁じられています。あなたは電子カルテの情報が不正閲覧される心配をしていますか（５段階評価を実施）。

Q24 あなたの病院内での経験をお聞きします。電子カルテなどで共有しないで欲しいとても機微な情報を、医療スタッフに話した経験はありますか。（単一回答）
１）ある　２）ない　３）その他

Q25 あなたは「共有しないで欲しいとても機微な情報を医療スタッフに話したことがある」と回答されました。あなたが話したその機微な情報の内容は、電子カルテに記載されていると思いますか（５段階評価を実施)。

Q26 あなたは「共有しないで欲しいとても機微な情報を医療スタッフに話したことがある」と回答されました。機微な情報を話した医療スタッフは、以下のどの職種ですか。1つ選んでください。機微な情報を話した経験が多い方は、一番多い職種を選択ください。
（単一回答）
１）医師　２）看護師　３）そのほかの医療従事者　４）病院の事務員　５）その他

Q27 診療の経過を電子カルテに記載する義務が医療スタッフにはあります。あなたの思うとても機微な情報を、どのように病院で共有して欲しいかを１つ選んでください。
（単一回答）
１）共有するスタッフの範囲は、病院が一定の規制をしてほしい
２）診療の経過で取得された情報なので、病院の全スタッフで共有しても良い
３）共有するスタッフの範囲は、あなた自身が決めたい
４）電子カルテに掲載しないでほしい
５）その他
６）わからない
